# In planta haploid induction by *kokopelli* mutants

**DOI:** 10.1093/plphys/kiad328

**Published:** 2023-06-10

**Authors:** Nathanaël M A Jacquier, Andrea R M Calhau, Yannick Fierlej, Jean-Pierre Martinant, Peter M Rogowsky, Laurine M Gilles, Thomas Widiez

**Affiliations:** Laboratoire Reproduction et Développement des Plantes, Univ Lyon, ENS de Lyon, UCB Lyon 1, CNRS, INRAE, Lyon F-69342, France; Limagrain, Limagrain Field Seeds, Research Centre, Gerzat F-63360, France; Laboratoire Reproduction et Développement des Plantes, Univ Lyon, ENS de Lyon, UCB Lyon 1, CNRS, INRAE, Lyon F-69342, France; Laboratoire Reproduction et Développement des Plantes, Univ Lyon, ENS de Lyon, UCB Lyon 1, CNRS, INRAE, Lyon F-69342, France; Limagrain, Limagrain Field Seeds, Research Centre, Gerzat F-63360, France; Laboratoire Reproduction et Développement des Plantes, Univ Lyon, ENS de Lyon, UCB Lyon 1, CNRS, INRAE, Lyon F-69342, France; Limagrain, Limagrain Field Seeds, Research Centre, Gerzat F-63360, France; Laboratoire Reproduction et Développement des Plantes, Univ Lyon, ENS de Lyon, UCB Lyon 1, CNRS, INRAE, Lyon F-69342, France

Dear Editors,

Double haploid (DH) technology is a powerful way to improve plant breeding efficiency (Jacquier et al. 2020). DH breeding based on in planta haploid induction represents an attractive way to induce haploid plants because it leads to seed-based haploid embryo formation, without the need of labor-intensive in vitro embryo rescue (Gilles, Martinant, et al. 2017). However, the availability of in planta haploid induction systems is presently limited to a handful of crop species (Jacquier et al. 2020). Research on the maize in planta haploid induction system allowed the identification of 3 main molecular players: *NOT-LIKE-DAD/MATRILINEAL/ZmPHOSPHOLIPASE-A1* (*NLD/MTL/ZmPLA1*) (Gilles, Khaled, et al. 2017; Kelliher et al. 2017; Liu et al. 2017), *DOMAIN OF UNKNOWN FUNCTION 679 MEMBRANE PROTEIN* (*ZmDMP*) (Zhong et al. 2019), and *PHOSPHOLIPASE D3* (*ZmPLD3*) (Li et al. 2021). Although the mode of action of maize haploid induction remains elusive, both *NLD/MTL/ZmPLA1* and *ZmPLD3* relate to lipid homeostasis (Gilles et al. 2021; Jacquier and Widiez 2021), whereas *ZmDMP* pinpoints links between haploid induction and fertilization defects (Jacquier et al. 2020, 2021). More precisely in *Arabidopsis*, the double mutant *Atdmp8/9* induces haploid embryos (Zhong et al. 2020) and shows fertilization defects with preferential single fertilization of the central cell (Takahashi et al. 2018; Cyprys et al. 2019). More recently, AtDMP8 and AtDMP9 were shown to be involved in acquisition of sperm cell fusion competence, thus playing a critical role in male / female gamete interaction (Wang et al. 2022). In order to identify additional genes exploitable for in planta haploid induction, we hypothesized that other mutants impaired in gamete interactions might also induce haploid embryos, similarly to what had been reported for the *Atdmp8/9* mutant. A search for genes that (i) were expressed in the *Arabidopsis* male gametophyte and (ii) known to lead to single fertilization events when mutated identified *AtKPL* (*KOKOPELLI*, AT5G63720) (Ron et al. 2010; Maruyama et al. 2013; Zhang, Maruyama, et al. 2023) as a candidate. In the present work, *Atkpl* mutant was shown to be able to trigger in planta maternal haploid induction.

In order to unambiguously detect maternal haploids in *Arabidopsis*, a female tester line was created combining both a recessive phenotypic marker, i.e. absence of trichomes due to the *glabra1* mutation, and an F1 hybrid genetic background between the Col-0 and L*er* accessions (*gl1*_Col/*gl1*_L*er*) (Fig. 1A). Haploid inducing capacity of *Atkpl* mutants was evaluated by the following pipeline (Fig. 1A): (i) Mutant pollen was deposited on pistils of the *gl1*_Col/*gl1*_L*er* tester line; (ii) the offspring were germinated and screened for absence of trichomes indicating either a putative desired maternal haploid seedling or an undesired self-pollination of the female tester line (contamination) (Fig. 1A); (iii) a first distinction between haploids seedlings and seedlings originated from contamination by self pollination of the tester female parent was based on two typical haploid phenotypes: sterility (absence of silique) and smaller organs (Fig. 1, A and B); and (iv) haploidy was further assessed by flow cytometry (Fig. 1, A and C) and (v) confirmed genetically using 83 single-nucleotide polymorphism (SNP) markers distributed on all 5 *Arabidopsis* chromosomes (Fig. 1A and Supplemental Table S2). All 83 SNPs are at a heterozygous state in the female tester line and are thus expected to behave like homozygous markers in maternal haploid plantlets, compared to diploid plantlets resulting of self-pollination expected to have high heterozygosity rates (Fig. 1, A and D).

**Figure 1.**
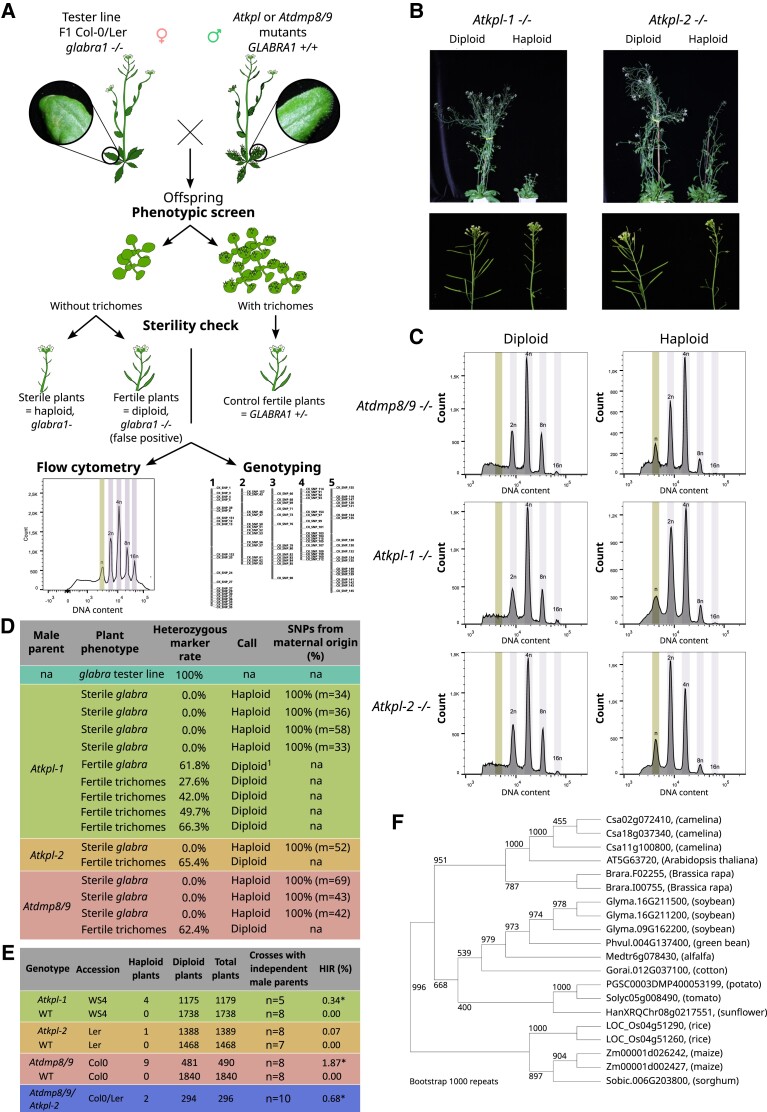
*Atkpl* triggers maternal haploid plants. **A)** Workflow used to assess maternal haploid induction. Pollen from mutants to be tested was used to pollinate the *glabra* (absence of trichomes) maternal tester line, which is a F1 hybrid between Col-0 and L*er* accessions. A first phenotypic screen based on absence of trichomes allowed selection of putative haploid plants, which were further validated using sterility criteria, flow cytometry, and genotyping of 83 SNP markers with genome-wide distribution. **B)** Representative pictures of diploid plants (left), as compared to the smaller haploid plants (right). **C)** Representative illustrations of ploidy verification of haploid and diploid sibling using flow cytometry for *Atkpl-1*, *Atkpl-2*, and *Atdmp8/9*. The *X* axes depict the DNA content for nuclei (DAPI fluorescence signal), whereas the *Y* axes represent the number of nuclei. **D)** Genotyping results to confirm haploidy and maternal origin of putative haploid plants (sterile *glabra*). m = numbers of markers for which it was possible to track unambiguously their parental origin.^1^ = example of diploid plant resulting from undesired selfing of the maternal tester line. na = non applicable. **E)** HIR of the different lines tested during the study. n = number of crosses. For each cross, emasculated inflorescences (1 to 4 siliques) were pollinated with a unique male parent. * *P* < 0.10, Wilcoxon signed-rank test. **F)** Phylogenetic tree of KOKOPELLI proteins in selected crops.

Two independent mutant T-DNA insertion lines for *AtKPL* were evaluated: *Atkpl-1* and *Atkpl-*2 in WS-4 and L*er* genetic backgrounds, respectively (Ron et al. 2010), and the *Atdmp8/9* double mutant (Col-0 background) was used as positive control for haploid induction (Supplemental Materials and Methods and Supplemental Table S1). Using the pipeline described above, the previously reported haploid induction capacity for *Atdmp8/9* double mutant (Zhong et al. 2020) was confirmed since 9 out of 490 plants screened were found to be haploid, i.e. a haploid induction rate (HIR) of 1.87% (Fig. 1E). Interestingly, haploid induction was also observed for both *Atkpl-1* and *Atkpl-2*, with 4 haploids among 1,179 plants (HIR 0.34%) and 1 haploid among 1,389 plants (HIR 0.07%), respectively (Fig. 1E). Since the 3 tested mutants (*Atdmp8/9*, *Atkpl-1*, and *Atkpl-2*) were in different genetic backgrounds, 3 different wild-type accessions, Col-0, WS-4, and L*er*, were evaluated as control for their HIR, and no haploids were scored out of 1840, 1,738, and 1,468 plants, respectively (Fig. 1E). Thus, the HIR of *Atkpl-1* is statistically significant in comparison to the HIR of WS-4 wild-type plants (Wilcoxon, *P*-value = 0.0056). All plants classified as haploids passed all steps of the pipeline. The final genetic analyses for haploid plants revealed that (i) all markers were detected as being at the “homozygous” state confirming the haploid status of those plants and (ii) 100% of SNPs that could be attributed to a parent came from the maternal parent (Fig. 1D). Thus, pollination using *Atkpl-1* and *Atkpl-2* mutant pollen produces maternal haploid plants.

Altogether, these results demonstrate that inactivation of *AtKPL* triggers in planta maternal haploid induction and that *Atkpl* mutants could be used as haploid inducer lines. The higher HIR of *Atkpl-1* (0.34%) as compared to *Atkpl-2* (0.07%) might come from difference in allele strength, since Ron et al. (2010) showed that *Atkpl-1* has more severely reduced seed set as compared to *Atkpl-2.* The fact that both the *Atdmp8/*9 double mutant and the *Atkpl* single mutant are impaired in double fertilization tends to support that single fertilization is an important feature for in planta haploid induction. Interestingly, a double mutation in the egg cell-specific endopeptidase genes *Atecs1/Atecs2* leads to preferential fertilization of the central cell (Jiang et al. 2022) and is able to confer haploid induction capacity (Mao et al. 2022; Zhang, Shi, et al. 2023). Contrary to *Atdmp8/*9 and *Atkpl*, *Atecs1/Atecs2* acts from the female side and thus reinforces the hypothesis that disruption of double fertilization, and probably preferential fertilization of the central cell over the egg cell, is a prerequisite for or a sufficient trigger of haploid induction. It would be interesting to test if combinations of these mutations have additive or synergistic effects on HIR. In order to start to address this question, the two *Atdmp8/*9 mutations were combined with the *Atkpl-*2 mutation. Aborted seeds and undeveloped ovules previously observed in siliques of the single mutants (Ron et al. 2010; Cyprys et al. 2019) were also observed for *Atdmp8/9/Atkpl-2* triple mutant (Supplemental Fig. S1). Evaluating haploid-inducing capacity of the *Atdmp8/9/Atkpl-2* triple mutant by the previously described pipeline, 2 haploids plants were detected among 296 plants (HIR 0.68%) (Figs. 1E and S2). Although the different genetic backgrounds of the *Atdmp8/9* (Col-0), *Atkpl-2* (L*er*), and triple mutant (mix) do not allow a definite conclusion, this result indicates that the combination of *Atdmp8/*9 with *Atkpl-2* likely does not have a synergistic effect on haploid induction in *Arabidopsis*.

Although the ∼0.34% HIR of *Atkpl-1* single mutant is quite low for an application in plant breeding, this may be different in other species. For example, maize *Zmdmp* mutants have a HIR of 0.15% compared to a HIR of ∼2% for *dmp* mutants in dicots. In addition, the possible synergy of the *kpl* mutation with other mutations needs to be explored. Indeed, although the maize *Zmdmp* single mutant has a very low HIR (∼0.15%), its combination with a mutation in *NLD/MTL/ZmPLA1* leads to a synergistic effect boosting the HIR 3 to 4 fold (Zhong et al. 2019; Jacquier et al. 2020). Taken together, both the per se evaluation of *kokopelli* mutants in other species and the interaction of *kpl* mutation with other mutations drive the interest in this new player in haploid induction. The identification of *AtKPL* orthologs in other species appears straight forward despite the presence of recent duplications in some species, as illustrated by the phylogenetic tree in Fig. 1F, which depicts some crop plants only. The presence of orthologous genes in both monocotyledonous and dicotyledonous plants can be thus leveraged for setting up or improving in planta haploid induction capacity in crops lacking this breeding tool.

## Supplementary Material

kiad328_Supplementary_Data

## Data Availability

The data supporting the findings of this study have been provided in the text and in the supplementary data files and are available upon request.

## Supplemental data

The following materials are available in the online version of this article.

**Supplemental Materials and Methods**. Materials and Methods.

**Supplemental Figure S1**. Phenotypes within siliques of selfed mutants.

**Supplemental Figure S2**. Identification of haploids in *Atdmp8/9/Atkpl-2* triple mutant.

**Supplemental Table S1**. Primers used in this study.

**Supplemental Table S2**. KASP markers used in this study to detect SNPs.
