# Supplementary material for: In planta haploid induction by kokopelli mutants
Source: Plant Physiol. 2023 Jun 10;193(1):182–5. doi: 10.1093/plphys/kiad328 (PMC10469538; doi:10.1093/plphys/kiad328)
Supplement: kiad328_Supplementary_Data [file kiad328_supplementary_data.pdf]

Supplemental data

In planta haploid induction by *kokopelli* mutants

Nathanaël M.A. Jacquier[1,2], Andrea R.M. Calhau[1], Yannick Fierlej[1], Jean-Pierre Martinant[2], Peter M. Rogowsky[1], Laurine M. Gilles[2], Thomas Widiez[1*]

[1] Laboratoire Reproduction et Développement des Plantes, Univ Lyon, ENS de Lyon, UCB Lyon 1, CNRS, INRAE, F-69342, Lyon, France.
[2] Limagrain, Limagrain Field Seeds, Research Centre, F-63360 Gerzat, France.
* corresponding author: Thomas Widiez (thomas.widiez@ens-lyon.fr)

Running title: *kokopelli* triggers haploid induction

Author contributions

Conceptualization: TW and NMAJ; Methodology: NMAJ and LMG; Formal Analysis: NMAJ; Investigation: NMAJ, ARMC, YF; Resources: TW, LMG and JPM; Writing – Original Draft: TW and NMAJ; Writing – Review & Editing: TW, NMAJ, PR, LMG, ARMC and YF; Visualization: NMAJ, Supervision: TW, LMG and JPM; Project Administration: TW and PR; Funding Acquisition: TW.

Supplemental Materials and Methods

Plant material

Arabidopsis thaliana Columbia-0 (Col0), Wassilewskija-4 (WS4), Landsberg erecta (Ler) accessions were used as wild type (WT) reference background. The insertion mutants were obtained from the Nottingham Arabidopsis Stock Center (NASC) (https://arabidopsis.info/) or from the INRAE Versailles T-DNA mutant collection (http://publiclines.versailles.inra.fr/) (Samson et al., 2002). The *Atkpl-1* (Versailles ID=FST 184H02, in WS4 background) and *Atkpl-2* (NASC_ID=N129764 in Ler background) mutant alleles were previously described (Ron et al., 2010). Mutant alleles for the *GLABRA1 (At3g27920*, *ATGL1*) gene were ordered in Col0 (*Atgl1-2*, NASC ID=N3126) and in Ler (*Atgl1-1*, NASC ID=N64) backgrounds. *Atgl1-1* and *Atgl1-2* were crossed and the resulted F1 hybrid plants were used as a *glabra* female tester lines to evaluate maternal haploid induction. The *A.thaliana* mutant for *dmp8/9* is in col0 background and were provided by Stefanie Sprunck (University of Regensburg, Germany) and described (Cyprys et al., 2019).

Genotyping of insertion mutant lines

T-DNA insertion for *Atkpl-1* was genotyped by PCR amplification of left border using KPL-1_RP and LB2_Versailles primers. Absence of T-DNA was verified by PCR amplification with KPL-1 and KPL_LP2 primers. The Ds transposable element insertion of *Atkpl-2* was genotyped by PCR amplification using KPL_LP2 and ds3-7 primers. Absence of T-DNA insertion was verified by PCR amplification using KPL_LP2 and AtKPL_RP (**Supplemental Table S1**). Genotyping of *Atdmp8/9* mutant was first performed using sanger sequencing (Cyprys et al., 2019) and then using KASP genotyping assay (**Supplemental Table S1**).

Plant growth conditions

The *Arabidopsis thaliana* plants were grown in soil under long-day (16h/8h light/dark cycle) conditions at 21°C and 60% humidity. For mutant selections, plants were grown horizontally on MS 1/2 medium (pH 5.7) (Duchefa), containing 0.8% Agar, (pH 5.7), in the absence of sucrose and supplemented with the appropriate antibiotic, with short day conditions for 10-13 days (8h/16h light/dark cycle) at 19°C and 60% humidity. Resistant plants were transplanted in soil and kept in short day conditions for 3 more weeks and genotyped using appropriate primers (**Supplemental Table S1**). Finally, plants were transferred to long day conditions and phenotyped.

Ploidy determination by flow cytometry

Youngest leaves from 5 to 7-week-old plants were harvested (2cm^2 in total). Leaves were finely chopped on glass Petri dishes in 500µl of fresh and cold Galbraith's buffer (GB) (Galbraith et al., 1983). Glass dishes were inclined to separate chopped leaves from the extract and additional 500µl of fresh and cold GB were added on chopped leaves to drag additional nuclei down. Extracts were filtered through 70µm, 40µm and 20µm nylon sieves on ice. Glass petri dishes and sieves were rinsed with 750µl of fresh and cold GB. Finally, 500µl of filtered extracts were transferred in polystyrene (Falcon® 5 mL Round-Bottom Polystyrene Tube, ref: 352052) tubes and 2.5µl of DAPI (5mg/ml) were added. Samples were directly

analyzed in LSR Fortessa flow cytometer operating at 640nm (Red laser) and 405nm (violet laser) using respectively APC (670/14) and DAPI (450/50) filters. Cleaning and rinsing steps were performed between each sample.

Verification of haploidization using genotyping

95 single nucleotide polymorphism (SNP) markers distributed along the 5 *A.thaliana* chromosomes and mainly discriminating between Col0, Ler and WS4 were selected and order to LGC Ltd. (Teddington, UK) (**Supplemental Table S2**) (Lutz and Schwechheimer, 2017). 83 out the 95 selected markers were found heterozygous in our *glabra*-/- F1 hybrid tester plants, constituting the final marker set (**Supplemental Table S2**). 5 leaf discs of 6mm diameter were extracted from putative haploid and control plantlets. Leaf discs were collected in plates with 96 deep wells and ground with two metallic balls (diameter 4 mm) in a 2010 Geno/Grinder® (SPEX Sample Prep, Stanmore, UK). Genomic DNA was extracted using a CTAB type lysis buffer and a magnetic bead-based purification. Competitive allele-specific PCR (KASP) assays were done flowing manufacturer instructions, LGC Ltd. (Teddington, UK), and as previously described (He et al., 2014).

Phylogenetic analysis

A list of putative orthologs of *Arabidopsis* KOKOPELLI proteins were identified through Hidden Markov Model (hmm) search tool available from phytozome platform (https://phytozome-next.jgi.doe.gov/) , and a subset was kept for the tree construction. The predicted amino acid sequences were aligned using MUSCLE in the SEAVIEW program (Gouy et al., 2010). Well-aligned amino acid blocks were selected for phylogenetic analyses using Gblocks. Maximum-likelihood phylogenetic analyses were performed in PhyML (Guindon et al., 2009) incorporating 1,000 bootstrap replicates and using LG substitution models for amino acid sequence data.

Statistical analysis

Haploid induction rates (HIR) were statistically compared using Wilcoxon signed-rank test with Rstudio. HIR resulted from test crosses done with independent male mutant plants were compared with their corresponding wild-type test crosses (**Figure 1e**). Wilcoxon test of *Atkpl-1* mutants compared to WS4, *Atkpl-2* mutants compared to Ler plants, *Atdmp8/9* mutants compared to Col0 plants, and *Atdmp8/9/ Atkpl-2* mutants compared to the pooled population of both Col0 and Ler wild-type plants resulted in the respective p-values: 0.0056; 0.4230; 0.0199 and 0.0875.

Supplemental Materials and Methods references:

Cyprys P, Lindemeier M, Sprunck S (2019) Gamete fusion is facilitated by two sperm cell-expressed DUF679 membrane proteins. Nature Plants **5**: 253–257

Galbraith DW, Harkins KR, Maddox JM, Ayres NM, Sharma DP, Firoozabady E (1983) Rapid flow cytometric analysis of the cell cycle in intact plant tissues. Science **220**: 1049–1051

Gouy M, Guindon S, Gascuel O (2010) SeaView Version 4: A Multiplatform Graphical User Interface for Sequence Alignment and Phylogenetic Tree Building. Mol Biol Evol **27**: 221–224

Guindon S, Delsuc F, Dufayard J-F, Gascuel O (2009) Estimating Maximum Likelihood Phylogenies with PhyML. *In* D Posada, ed, Bioinformatics for DNA Sequence Analysis. Humana Press, pp 113–137

He C, Holme J, Anthony J (2014) SNP Genotyping: The KASP Assay. *In* D Fleury, R Whitford, eds, Crop Breeding. Springer New York, pp 75–86

Lutz U, Schwechheimer C (2017) A set of Columbia-0-specific single nucleotide polymorphism markers for the genetic analysis of natural variation in *Arabidopsis thaliana*. doi: 10.1101/153197

Ron M, Saez MA, Williams LE, Fletcher JC, McCormick S (2010) Proper regulation of a sperm-specific cis-nat-siRNA is essential for double fertilization in Arabidopsis. Genes Dev **24**: 1010–1021

Samson F, Brunaud V, Balzergue S, Dubreucq B, Lepiniec L, Pelletier G, Caboche M, Lecharny A (2002) FLAGdb/FST: a database of mapped flanking insertion sites (FSTs) of Arabidopsis thaliana T-DNA transformants. Nucleic Acids Research **30**: 94–97

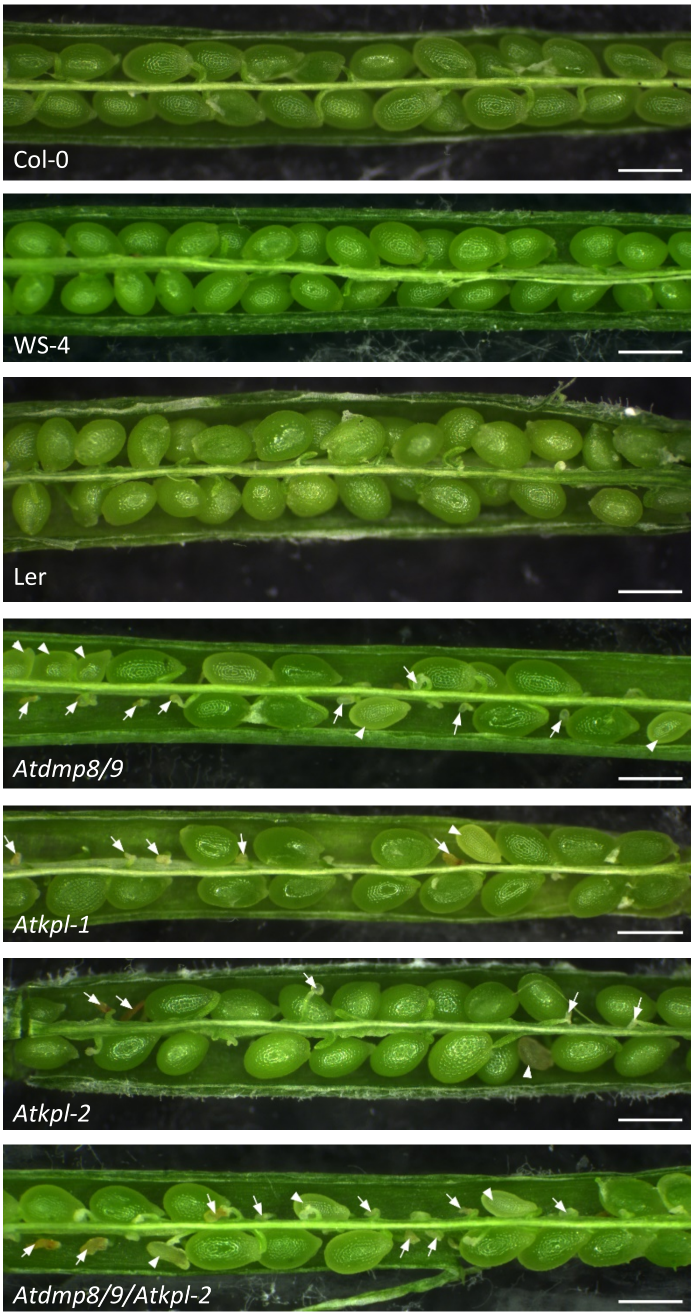

Supplemental Figure S1. Phenotypes within siliques of selfed mutants.

Aborted seeds (arrows) and undeveloped ovules (arrowheads) were detected in siliques of selfed *Atkpl-1, Atkpl-2, Atdmp8/9* and *Atdmp8/9/Atkpl-2* mutants in comparison to wild type accessions (Scale bars, 50 µm).

(a)

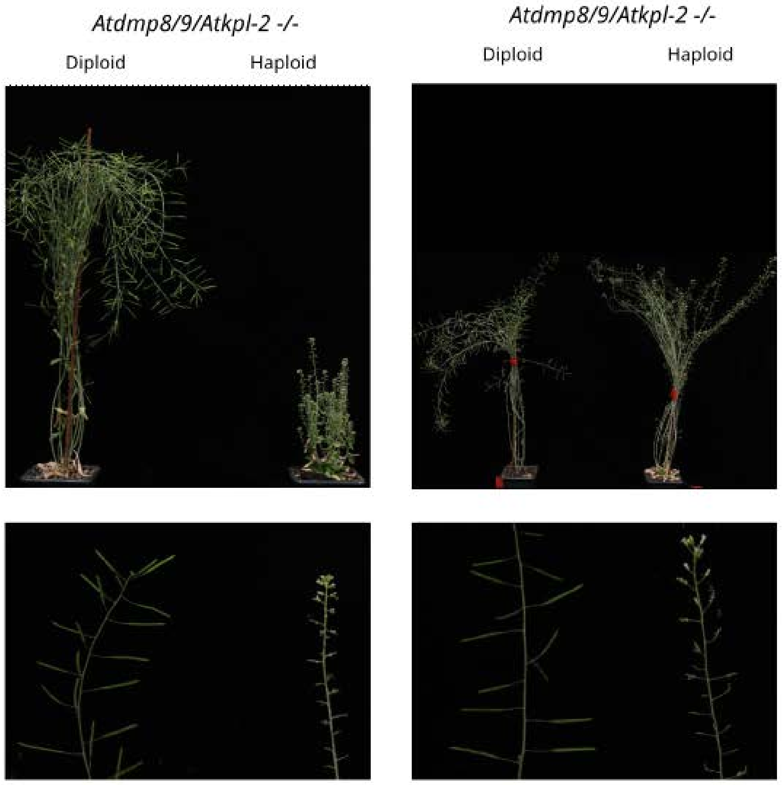

(b)

Male parent	Plant phenotype	Heterozygous marker rate	Call	SNPs from maternal origin (%)
na	*glabra* tester line	100%	na	na
Atdmp8/9/ Atkpl-2	Sterile *glabra*	0.0%	Haploid	100% (m=34)
	Sterile *glabra*	0.0%	Haploid	100% (m=40)
	Fertile *glabra*	35.6%	Diploid	na
	Fertile *glabra*	51.1%	Diploid	na
	Fertile *glabra*	62.2%	Diploid	na
	Fertile *glabra*	60.0%	Diploid	na
	Fertile trichome	56.7%	Diploid	na
	Fertile trichome	62.2%	Diploid	na
	Fertile trichome	56.7%	Diploid	na
	Fertile trichome	34.4%	Diploid	na
	Fertile trichome	56.7%	Diploid	na
	Fertile trichome	37.8%	Diploid	na
	Fertile trichome	37.8%	Diploid	na
	Fertile trichome	51.1%	Diploid	na
	Fertile trichome	53.3%	Diploid	na
	Fertile trichome	48.9%	Diploid	na
	Fertile trichome	54.4%	Diploid	na

<u>Supplemental Figure S2</u>. Identification of haploids in *Atdmp8/9/Atkpl-2* triple mutant.

(**a**) Diploid plants (left), as compared to sterile haploid plants (right) obtained using the *Atdmp8/9/Atkpl-2* triple mutant as male parent on female *glabra* tester line. (**b**) Genotyping results to confirm haploidy and maternal origin of putative haploid plants (sterile *glabra*). m= numbers of markers for which it was possible to track unambiguously their parental origin. The "fertile *glabra*" plants are examples of diploid plants resulting from undesired selfing of the maternal tester line. na= non-applicable.

Supplemental Table S1: Primers used in this study

Primer name	Sequence 5'-3'
KPL_LP2	CCAACTTCACACGTCCTCTTTTGGG
AtKPL_RP	GTCTGCGACAGGTTCTGAGTC
KPL-1_RP	GCTTCCCACGAACACTTTGTTACAC
LB2_Versailles	CAACCCTCAACTGAAACGGGCCGGA
ds3-7	GTTACCGACCGTTTTCATCC

KASP Assay ID	sequence context
AtDMP8_insC	TCGTCTCGGTCGCCCAAACCTGTCCCTCTCTCTTCACTGCCTATGCTTCC[/C]GGCAGGAGCCGCCGCGGGAGGAGGAAAAGGTAGAAAACGTCGCATGGTGG
AtDMP9_insG	CCTTCTCGTTCACCAAAACCCGTCTTAATCTCTTCATTGCCTTCCCTCCC[/G]GTCAGGAGCCGCCGCTGGAGGAGGAAGAGGTCGAAAACGTCGCATGGTGG

Supplemental Table S2: KASP markers used in this study to detect SNPs

*KASP Maker ID that could be directly ordered through LGC Genomics and described in Lutz and Schwechheimer (2017).

$ Verification of marker status in the *glabra* F1 hybrid (gl1_Col/gl1_Ler) tester line

LGC_SNP_name*	Chromosome	Position	Related locus	Col-0	Ws-2	Ler-0	Marker at heterozygous state in tester line $
CK_SNP_1	1	151639	AT1G01400	A	T	T	yes
CK_SNP_3	1	1503744	AT1G05190	A	G	G	yes
CK_SNP_4	1	2486946	AT1G08010	A	T	T	yes
CK_SNP_5	1	3245682	AT1G09960	T	T	G	yes
CK_SNP_7	1	4798100	AT1G14000	G	A	A	No
CK_SNP_8	1	5588488	AT1G16340	G	A	A	yes
CK_SNP_12	1	8712855	AT1G24577	T	C	C	yes
CK_SNP_13	1	9517294	AT1G27410	A	G	G	yes
CK_SNP_14	1	10277615	AT1G29360	C	T	C	No
CK_SNP_15	1	11054350	AT1G29360	T	A	A	No
CK_SNP_20	1	18100755	AT1G48920	A	A	G	yes
CK_SNP_24	1	22037143	AT1G59870	A	G	G	yes
CK_SNP_27	1	24367754	AT1G65550	C	T	T	yes
CK_SNP_29	1	26732602	AT1G70900	T	T	A	yes
CK_SNP_30	1	27514348	AT1G73170	T	T	C	yes
CK_SNP_31	1	28268857	AT1G75335	C	T	T	yes
CK_SNP_33	1	29053148	AT1G77310	G	A	A	yes
CK_SNP_34	1	29861728	AT1G79380	A	G	G	yes
CK_SNP_35	1	30349862	AT1G80750	T	C	C	yes

CK_SNP_36	1	28951797	AT1G77060	C	T	T	yes
CK_SNP_37	1	25958411	AT1G69040	A	T	T	yes
CK_SNP_39	1	5343813	AT1G15540	A	C	C	yes
CK_SNP_151	1	7912076	AT1G22410	T	A	A	yes
CK_SNP_152	1	17307886	AT1G47230	T	C	C	yes
CK_SNP_43	2	1666106	AT2G04760	A	G	G	yes
CK_SNP_46	2	6364468	AT2G14830	G	A	A	yes
CK_SNP_47	2	7144270	AT2G16485	A	T	T	yes
CK_SNP_50	2	9506650	AT2G22400	A	G	G	yes
CK_SNP_51	2	10231013	AT2G24060	C	T	T	yes
CK_SNP_52	2	11070503	AT2G25950	T	A	A	yes
CK_SNP_53	2	11855191	AT2G27810	T	C	C	yes
CK_SNP_56	2	14184854	AT2G33490	T	C	C	yes
CK_SNP_57	2	14983485	AT2G35637	T	C	C	yes
CK_SNP_61	2	18101154	AT2G43650	G	A	A	yes
CK_SNP_62	2	18880409	AT2G45880	G	A	A	yes
CK_SNP_63	2	19608670	AT2G47890	G	C	C	yes
CK_SNP_157	2	1166219	AT2G03820	T	C	C	yes
CK_SNP_66	3	1692799	AT3G05725	T	A	A	yes
CK_SNP_68	3	3241867	AT3G10420	T	C	C	yes
CK_SNP_69	3	4021191	AT3G12670	G	A	G	yes
CK_SNP_71	3	5580150	AT3G16420	T	C	C	yes
CK_SNP_73	3	7148614	AT3G20475	A	C	C	yes
CK_SNP_76	3	9493272	AT3G25930	T	A	A	yes
CK_SNP_79	3	15018944	AT3G42960	G	T	T	yes
CK_SNP_80	3	15758769	AT3G43920	A	G	G	yes
CK_SNP_82	3	17314526	AT3G47000	C	T	T	yes
CK_SNP_83	3	18104656	AT3G48830	T	G	G	yes
CK_SNP_84	3	18880548	AT3G50790	T	C	T	yes
CK_SNP_85	3	19665585	AT3G53040	C	T	T	yes
CK_SNP_87	3	21231006	AT3G57380	A	G	A	No
CK_SNP_90	3	23371626	AT3G63250	G	C	C	yes
CK_SNP_91	4	889684	AT4G02020	A	A	G	yes
CK_SNP_92	4	1772648	AT4G03820	C	C	A	yes
CK_SNP_93	4	2662255	AT4G05150	C	C	A	yes
CK_SNP_97	4	7162842	AT4G11920	C	G	G	yes
CK_SNP_99	4	8717065	AT4G15270	C	C	T	yes
CK_SNP_101	4	10297657	AT4G18710	G	T	T	yes
CK_SNP_103	4	11862501	AT4G22540	C	C	T	yes
CK_SNP_104	4	12631344	AT4G24430	C	G	G	yes
CK_SNP_105	4	13546672	AT4G26970	C	G	G	yes
CK_SNP_107	4	14981906	AT4G30750	T	C	C	yes
CK_SNP_109	4	16532462	AT4G34610	C	T	T	yes
CK_SNP_112	4	18489502	AT4G39850	A	G	G	yes
CK_SNP_114	4	482152	AT4G01120	T	T	G	yes
CK_SNP_115	4	12992095	AT4G25420	A	T	T	yes
CK_SNP_154	4	6397843	AT4G10320	A	G	G	yes

CK_SNP_159	4	17040794	AT4G36010	T	A	A	yes
CK_SNP_160	4	17488368	AT4G37120	T	C	C	yes
CK_SNP_117	5	909803	AT5G03590	T	C	C	No
CK_SNP_119	5	2463615	AT5G07740	A	G	G	yes
CK_SNP_120	5	4017317	AT5G12400	C	G	G	yes
CK_SNP_121	5	4806180	AT5G14860	A	G	G	yes
CK_SNP_124	5	7159157	AT5G21080	T	G	G	yes
CK_SNP_125	5	7928198	AT5G23510	C	G	G	yes
CK_SNP_127	5	10281139	AT5G28290	G	A	A	No
CK_SNP_128	5	13637129	AT5G35410	T	G	G	yes
CK_SNP_130	5	14986369	AT5G37730	G	C	C	yes
CK_SNP_131	5	15749648	AT5G39340	G	G	T	No
CK_SNP_132	5	16535978	AT5G41320	C	T	T	yes
CK_SNP_134	5	18100380	AT5G44830	T	C	T	yes
CK_SNP_135	5	18880655	AT5G46540	T	A	A	yes
CK_SNP_138	5	21287079	AT5G52440	A	A	T	yes
CK_SNP_139	5	22011670	AT5G54220	C	A	A	yes
CK_SNP_141	5	23783404	AT5G58900	C	A	A	yes
CK_SNP_142	5	24357567	AT5G60590	A	G	G	yes
CK_SNP_143	5	25164679	AT5G62670	G	A	A	yes
CK_SNP_145	5	26676209	AT5G66810	T	T	C	yes
CK_SNP_147	5	3183429	AT5G10150	A	T	T	yes
CK_SNP_149	5	21051069	AT5G51800	T	C	C	yes
CK_SNP_155	5	105427	AT5G01260	C	T	T	yes
